# Molecular Characterization of *Sarcocystis* Species Isolated from Sheep and Goats in Riyadh, Saudi Arabia

**DOI:** 10.3390/ani9050256

**Published:** 2019-05-21

**Authors:** Dina M. Metwally, Mashael A. Al-Damigh, Isra M. Al-Turaiki, Manal F. El-Khadragy

**Affiliations:** 1 Zoology Department, Faculty of Science, King Saud University, Riyadh 11451, Saudi Arabia; manalelkhadragy@yahoo.com; 2 Parasitology Department, Faculty of Veterinary Medicine, Zagazig 44519, Egypt; 3 Biology Department, Faculty of Education, Al-Majmaah University, Al-Majmaah 11952, Saudi Arabia; ma.aldamigh@gmail.com; 4 Department of Information Technology, Faculty of Computer and Information Science, King Saud University, Riyadh 11451, Saudi Arabia; ialturaiki@ksu.edu.sa; 5 Department of Zoology and Entomology, Faculty of Science, Helwan University, Cairo 11795, Egypt

**Keywords:** *Sarcocystis* species, COX1, sheep and goats

## Abstract

**Simple Summary:**

In this article, we investigated the occurrence of *Sarcocystis* species in samples of sheep and goats obtained from slaughterhouses in Riyadh, Saudi Arabia. We searched for tongue, heart, esophagus, diaphragm and skeletal muscle tissues. Fragments of these tissues were investigated by macroscopic evaluations, direct optical microscopy of tissue fragments, optical microscopy of digested fragment sediment, transmission electron microscopy and PCR followed by nucleotide sequencing. From the set of information obtained, we searched 230 sheep, and 84 goats; 91 and 36 were found to be infected, respectively. Transmission electron microscopy (TEM) revealed *Sarcocystis tenella* (*S. tenella*) in sheep and *Sarcocystis capracanis* (*S. capracanis*) in goats. *Sarcocystis* species were confirmed in Saudi Arabian sheep and goats by molecular testing. *S. capracanis* was most closely related to *S. tenella*, with the mitochondrial cytochrome c oxidase subunit I gene (COX1) sequences sharing a (91.7%) identity.

**Abstract:**

Sarcocystosis is induced by species of *Sarcocystis*, which is an intracellular protozoan parasite in the phylum Apicomplexa. The diversity and importance of *Sarcocystis* species in sheep and goats in Saudi Arabia are poorly understood. In this study, the tongue, esophagus, heart, diaphragm, and skeletal muscles were collected from 230 sheep and 84 goats, and the tissues were examined for the presence of *Sarcocystis* species by macroscopic examination and light microscopy. Microscopic *Sarcocystis* species cysts were found in both sheep and goats. Transmission electron microscopy (TEM) revealed *S. tenella* in sheep and *S. capracanis* in goats. *Sarcocystis* species were confirmed for the first time in Saudi Arabian sheep and goats by molecular testing. *S. capracanis* was most closely related to *S. tenella,* with the COX1 sequences sharing 91.7% identity. A phylogenetic analysis produced similar results and indicated that the *Sarcocystis* isolates were within a group of *Sarcocystis* species in which dogs were the final host. Finally, the *Sarcocystis* species cysts from sheep and goats could be grouped together, indicating that they were strongly related.

## 1. Introduction

*Sarcocystis* species have an obligatory heteroxenous (prey-predator) life cycle [1]. Sarcocystis infection is common among various vertebrates, including people [2,3,4,5,6,7]. Sheep are intermediate hosts for at least six species, including *S. tenella*, *S. arieticanis*, *S. gigantea*, *S. medusiformis*, *S. mihoensis*, and *S. microps*, which are morphologically differentiated based on their sarcocyst wall ultrastructure. *Sarcocystis tenella* and *S. arieticanis* produce microscopic sarcocysts transmitted by canids, while *S. gigantea* and *S. medusiformis* produce macroscopic cysts transmitted by felids [2]. The remaining two species, *S. mihoensis* and *S. microps*, are transmitted by canids and are unusual or rare species of *Sarcocystis*. *Sarcocystis mihoensis*, reported only from Japan, and they produce macroscopic sarcocysts [8,9]. Goats can be infected by three different *Sarcocystis* species: *S. capracanis* and *S*. *hircicanis*, in which dogs are the final host, and *S*. *moulei*, in which cats are the final host [10,11]. In Saudi Arabia, sheep and goats are used for both meat and milk production. There are three main breeds of sheep. Niemy and Najdy are local sheep, while the Sawakny breed is imported from Sudan and is used for meat production only [12]. Previous studies have examined the prevalence of *Sarcocystis* in Najdy sheep and camels slaughtered in Riyadh [13,14], *Sarcocystis* in slaughtered camels in the Al-Ahssa region [15], and *Sarcocystis* parasites in the muscles of Arabian deer using optical and electron microscopy techniques [16]. In recent years, molecular techniques have been used as an epidemiological and diagnostic tool to determine which *Sarcocystis* species are involved in an infection [17,18]. Molecular characterization using appropriate markers has become an essential tool for accurate identification of *Sarcocystis* species and for research regarding the phylogenetic relationships of these species. As for *Sarcocystis* species in cervids, nucleotide sequences of the nuclear 18S ribosomal RNA gene (18S rRNA) and/or the mitochondrial cytochrome c oxidase subunit I gene (COX1) of approximately 30 species are currently available for comparative molecular studies [19,20]. Of these markers, COX1 has been found to be superior to 18S rRNA in resolving unclear species boundaries of closely related *Sarcocystis* species in various ruminant intermediate hosts [20,21,22]. The present study aimed to analyze the genes encoding COX1 in *Sarcocystis* species isolated from naturally infected sheep and goats in Riyadh, Saudi Arabia.

## 2. Materials and Methods

### 2.1. Sample Collection

The Institutional Committee of Postgraduate Studies and Research at King Saud University (Saudi Arabia) approved this study. Tissue samples were collected by veterinarians during postmortem inspections of slaughtered animals performed at the Al-Sada Abattoir in Riyadh, Saudi Arabia, from March 2016 to January 2017. Tissue samples were isolated from 230 sheep (including the Najdy, Niemy, and Sawakny breeds) and 84 goats. The entire tongue, heart, skeletal muscle, diaphragm, and esophagus were collected from each animal and individually stored in sealed plastic bags. The tissues were then transported to the laboratory in boxes containing ice packs. 

### 2.2. Macroscopic Analysis

A macroscopic analysis was performed on the same day as tissue collection. Five transverse cuts were performed using a scalpel on the tongue and heart to reveal the macroscopic cysts. The entire esophagus was longitudinally sectioned to expose its lumen, and its internal and external walls were analyzed macroscopically [23].

### 2.3. Microscopic Analysis

The microscopic analysis for cysts using fresh tissues was performed using a squash preparation [24]. Separate fragments of each tissue measuring approximately 5 mm thick were firmly squashed between two slides and examined under an optical microscope at 40× and 100× magnifications. This procedure was performed in triplicate for each tissue. *Sarcocystis* species cysts were processed for light microscopy (LM), transmission electron microscopy (TEM), and DNA analysis.

### 2.4. Digestion Method

Approximately 20 g of each tissue was minced and then digested for 30 min at 37 °C in 100 mL of digestion medium containing 1.3 g of pepsin, 3.5 mL HCl, and 2.5 g NaCl in 500 mL of distilled water [25]. After digestion, the mixture was centrifuged for 3 min at 3500× *g*, and then the sediment was stained with Giemsa and examined by optical microscopy at 400× magnification [26].

### 2.5. Transmission Electron Microscopy (TEM)

*Sarcocystis* species cysts were collected, fixed in 2% glutaraldehyde solution in 0.1 M sodium cacodylate buffer (pH 7.4) for 2 h at 15–25 °C, and stored at 4 °C until processing. After fixation, the samples were washed in 0.1 M sodium cacodylate buffer (pH 7.4), fixed with 1% osmium tetroxide, dehydrated in different acetone solutions (30, 40, 50, 70, 90, and 100%), and treated in blocking buffer with 1% phosphotungstic acid and 1% uranyl acetate. Next, the 100% acetone solution was replaced with Polybed resin, followed by paraffin embedding and polymerization in an oven at 60°C. Semithin cuts were made to observe the *Sarcocystis* species cysts by light microscopy. Ultrathin sections were stained with uranyl acetate and lead citrate and then examined using a JEM100-CX transmission electron microscope at 80 kV. [23].

### 2.6. Molecular Analysis

#### 2.6.1. DNA Extraction and PCR Amplification

Small pieces from each individual microscopic *Sarcocystis* isolate were washed five times with sterile distilled water, and genomic DNA (gDNA) was extracted using the QIAamp DNA mini kit for tissue and blood according to the manufacturer’s instructions (Qiagen GmbH, Hilden, Germany Cat. No. 51304). The mitochondrial COX1 gene was amplified using specific primer pairs (Table 1) [21,22]. Amplification was carried out in a thermocycler (Veriti^®^96-well thermal cycler, model 9902, Biosystem) in a 20-μL reaction mixture containing 4 μL of master mix (5×), 12.8 μL of RNase-free water, 1.2 μL of both forward and reverse primers, and 2 μL of DNA template. The PCR program consisted of an initial denaturation step at 94 °C for 5 min, followed by 40 cycles of denaturation at 94 °C for 45 seconds, annealing at 50 °C for 45 seconds, and a final extension step at 72 °C for 10 min. The PCR products were analyzed by 1% agarose gel electrophoresis.

#### 2.6.2. DNA Sequencing and Phylogenetic Analysis

The PCR products derived from the target COX1 gene were purified and subjected to sequencing with both forward and reverse complements on the Genetic Analyzer at the Central Lab of King Saud University. The sequences were analyzed using *Geneious* software version 11.1.4. All sequences were slightly truncated using an error probability method with a limit of 0.05 at both ends. A BLAST search was performed for each sequence to find related sequences. 

The neighbor-joining method was used with the Tamura Nei model in order to generate a phylogenetic tree [27]. The bootstrap method was used for resampling with the number of replicates set to 1000.

### 2.7. Statistical Analysis

Statistical analysis was performed using the Statistical Package for Social Sciences (SPSS) software (version 17, SPSS, Inc., Chicago, IL, USA). Continuous and categorical variables were displayed as the means ± standard deviation (SD) and percentages, respectively. Differences in the prevalence between males and females and different age groups were analyzed by a Chi-square test (χ^2^). *p* Values < 0.05 were considered statistically significant. The lengths and widths of at least ten cysts from each organ (heart, tongue, and esophagus) were determined by light microscopy and expressed as the mean sizes and amplitudes of variation.

## 3. Results

### 3.1. Prevalence of Natural Infection

An investigation of random muscles samples obtained from 314 slaughtered sheep and goats for the presence of *Sarcocystis* species revealed that the prevalence of *Sarcocystis* in naturally infected Niemy sheep, Najdy sheep, Sawakny sheep, and goats was 35.18%, 43.33%, 43.75%, and 42.85%, respectively (Table 2).

The prevalence of *Sarcocystis* species in young and adult male sheep was 43.53% and 35.84%, respectively. The prevalence of *Sarcocystis* species was 42.85% and 33.33%, respectively, in young and adult female sheep. In young and adult male goats, the prevalence was 47.45% and 40%, respectively. Finally, the prevalence in young female goats was 35.29%, whereas the examined adult female goats were not infected (Table 3).

The distribution of *Sarcocystis* cysts varied from one sheep organ to another, and from one sheep breed to another. The distribution was different than that in goats, but in general, the diaphragm and skeletal muscle showed the highest infection levels (Niemy sheep, 84.21%; Najdy sheep, 61.53%; Sawakny sheep, 85.71%; goats, 61.11%) of all the organs examined. The tongue was the organ with the lowest infection rate (Najdy sheep, 12.82%; Sawakny sheep, 21.42%; goats, 19.44%), with the exception of Niemy sheep, in which the esophagus had the lowest infection rate (28.94%) (Table 4).

### 3.2. Morphological Characteristics of the Cysts

An analysis of the esophagus, tongue, diaphragm, skeletal muscle, and heart revealed microscopic *S*. *tenella* and *S*. *capracanis* cysts in sheep and goats, respectively. Macroscopic cysts were not recorded in the present study.

#### 3.2.1. *S. tenella* Cysts

**Figure 1:**
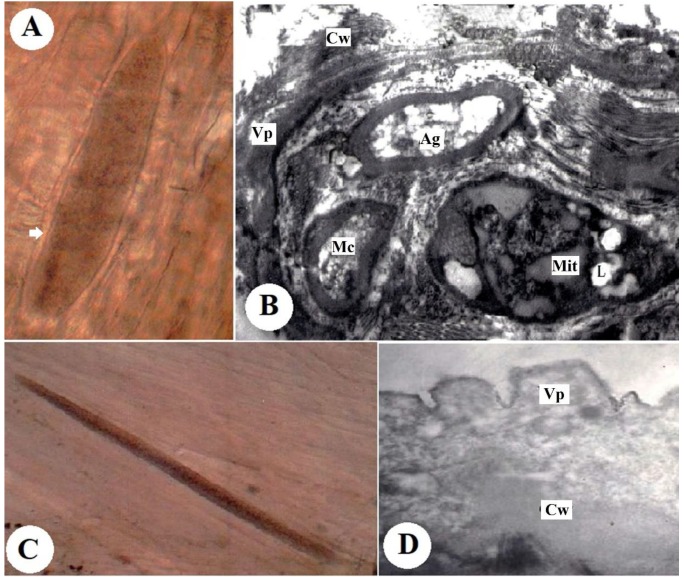
(**A**) Fresh cyst of *S. tenella* in the muscle of naturally infected sheep (digital camera). (**B**) Ultrastructure of *S. tenella* with a fully formed cyst wall (Cw) showing finger-like villar projections (Vp) (×16,000). (**C**). Fresh cyst of *S. capracanis* in the muscle of naturally infected goats (digital camera). (**D**) Ultrastructure of *S. capracanis* with the fully formed cyst wall (Cw) showing finger-like villar projections (Vp), metrocytes (Mc), lipid (L), amylopectin granules (Ag) and mitochondria (Mit) (×6300).

The fresh microscopic cysts were spindle-shaped with fully formed walls. They measured 851 µm × 75 µm to 185 µm × 31 µm (Figure 1A). An ultrastructure analysis of the cyst wall revealed that it was 0.74-µm thick and consisted of finger-like villar projections (Vp). The rim of the Vp consisted of minute undulations over the basal third. The microtubules were often arranged in bundles (Figure 1B).

#### 3.2.2. *S. capracanis* Cysts

The fresh microscopic cysts had a spindle shape with a thin wall and measured 869 µm × 648 µm to 370 µm × 70 µm (Figure 1C). The ultrastructural analysis showed that the cyst wall had palisade-like Vp with long constrictions at the base (Figure 1D).

### 3.3. Molecular Analysis

Partial amplification of the COX1 gene (1085 bp) is shown in all DNA samples of microscopic *Sarcocystis* species.

### 3.4. Molecular Characterization of the COX1 Gene

A total of 23 samples, including seven Sawakny samples, nine Najdy samples, two Naimy samples, and five goat samples, were examined. The samples were 1034-1106 nucleotides long and shared a 91.7% pairwise identity.

**Figure 2:**
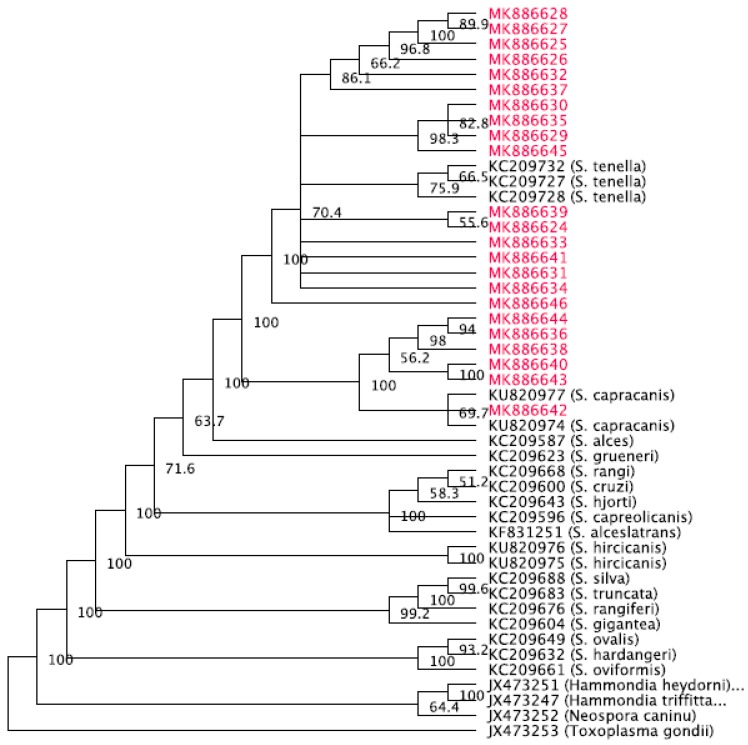
Phylogenetic tree of the 23 samples along with several sequences published in GenBank.

The phylogenetic tree was generated with *Toxoplasma gondii*, accession number JX473253, as the out-group. The phylogenetic tree with consensus support values is shown in Figure 2. The samples form a clade with *S. tenella* sequences given by accession numbers KC209727, KC209728, and KC209732 from Norwegian sheep. In addition, the clade contains *S. capracanis* sequences with accession numbers KU820974 and KU820977 from Chinese goats.

## 4. Discussion

To determine the presence of *Sarcosystis* spp., the present study examined the esophagus, tongue, diaphragm, skeletal muscle, and heart tissue from 230 sheep and 84 goat specimens. It was concluded that the overall prevalence for the *Sarcosystis* spp. were as follows: Sheep specimens, 39.5%, and goat specimens, 42.8%. It was unexpected that adult animals had a lower prevalence *Sarcosystis*; this could be due to low number of adult animal samples. Microscopic *Sarcocystis* species cysts were detected in the current work by means of tissue squash, pepsin-hydrochloric acid digestion, and Hematoxylin and eosin stain. The tissue squash method was a fast and practical diagnostic tool and detected a higher number of positive animals than the pepsin-hydrochloric acid digestion technique, although these methods did not permit differentiation among the *Sarcocystis* species. There was great variation in the lengths and widths of the observed cysts, which may be related to the age of the cysts.

As mentioned previously, the only documented evidence of *Sarcosystis* species in Saudi Arabia was based on TEM [12,28]. The present study included the separation of *S. arieticanis* cysts. In all previous studies, the absence of septa in *S. arieticanis* cysts [29] was probably due to the low number of cysts examined (n = 3). Previous reports throughout the world have recorded *S. tenella* infection in sheep from Brazil [18], Tunisia [30], and Iraq [31]. The cysts examined by TEM in the goat tissues in the present study had wall morphological characteristics of *S. capracanis*, with finger-like or palisade Vp [32]. The morphology of *S. capracanis* observed in the present study conforms to the TEM classification by [33] and was similar to the morphology of *S. capracanis* in domestic goats observed in Japan [34], the Philippines [35], Egypt [36], and China [37]. In addition, similar sarcocyst morphology has been described from other domestic ruminant animals, such as *S. tenella* in domestic sheep [38]. In the present study, certain DNA samples from the microscopic *Sarcocystis* species yielded no bands during the partial PCR amplification of the COX1 gene. This may be due to the low number of COX1 gene copies. Therefore, it is essential to utilize additional PCR cycles in these studies. When these sequences were queried via Basic Local Alignment Search Tool (BLAST), the sequence of COX1 from *S. tenella* (18 sheep) shared high identity (91.7%) with that from *S. capracanis* (5 goats). The high identity between the COX1 sequences from *S. tenella* and *S. capracanis* suggests that sheep and goats can harbor the same *Sarcocystis* species [39].

Since this study demonstrated a high prevalence of *Sarcocystis* infection by microscopic cyst forming species, sheep and goats could be considered alternative intermediate hosts for *S. tenella* and *S. capracanis*. The complete sequences of the gene that encoded COX1, or the sequence analysis of other genetic loci, are essential in order to understand the genetic diversity found across the world among *Sarcocystis* species. Our data collected in this study highlight the need to carry a more in-depth phylogenetic analysis and study of *Sarcocystis* species with more taxa and different molecular markers. Because of its high variance, studies with the internal transcribed spacer gene 1 (ITS-1) region should be performed to further distinguish among the closely related *Sarcocystis* species.

## Figures and Tables

**Table 1:** List of primers used for the amplification of genes encoding c oxidase subunit I gene (COX1) in *Sarcocystis* species.

Genes	Primers	Sequences	Fragment Size	References
*COX1*	SF1	ATGGCGTACAACAATCATAAAGAA	1100 bp	[21,22]
	SR9	ATATCCATACCRCCATTGCCCAT		

**Table 2:** Prevalence of *Sarcocystis* species in sheep and goats.

Animals	No. Examined (%)	No. Infected (%)
**Sheep**	Niemy 108	38 (35.18%)
	Najdy 90	39 (43.33%)
	Sawakny 32	14 (43.75%)
	230	91 (39.56%)
**Goats**	84	36 (42.85%)
**Total**	314	127 (40.44%)

**Table 3:** Prevalence of *Sarcocystis* species among different categories of sheep and goats.

Categories	Sheep		Goats	
	No. Examined	No. Infected (%)	No. Examined	No. Infected (%)
**Young males (under 1 year)**	147	64 (43.53%)	59	28 (47.45%)
**Adult males (above 1 year)**	53	19 (35.84%)	5	2 (40%)
**Young females (under 1 year)**	21	9 (42.85%)	17	6 (35.29%)
**Adult females (above 1 year)**	9	3 (33.33%)	3	0 (0%)
**Total**	230	95 (41.30%)	84	36 (42.85%)

**Table 4:** Organ distribution of *Sarcocystis* species in sheep and goats.

Animals	No. Infected	Esophagus	Tongue	Diaphragm	Heart	Skeletal Muscles
		No. (%)	No. (%)	No. (%)	No. (%)	No. (%)
**Niemy**	38	11	14	26	23	32
		(28.94%)	(36.84%)	(68.42%)	(60.52%)	(84.21%)
**Najdy**	39	8	5	24	14	17
		(20.51%)	(12.82%)	(61.53%)	(35.89%)	(43.58%)
**Sawakny**	14	5	3	12	8	11
		(35.71%)	(21.42%)	(85.71%)	(57.14%)	(78.57%)
**Goats**	36	9	7	21	17	22
		(25%)	(19.44%)	(58.33%)	(47.22%)	(61.11%)
**Total**	127	33	29	83	62	82
		(25.98%)	(22.83%)	(65.35%)	(48.81%)	(64.56%)
